# Thrombocytopenia-Absent Radius (TAR): Case report of dental implant and surgical treatment

**DOI:** 10.4317/jced.56642

**Published:** 2020-12-01

**Authors:** Danilo-Viegas da Costa, Vania-Eloisa de Araújo, Fernando-Antônio-Mauad de Abreu, Giovanna-Ribeiro Souto

**Affiliations:** 1 DDS, MDSc student. Department of Dentistry. Pontifical Catholic University of Minas Gerais; 2 DDS, MDSc, PhD. Department of Dentistry. Pontifical Catholic University of Minas Gerais

## Abstract

Thrombocytopenia-absent radius (TAR) syndrome is a congenital malformation in which affected individuals present reductions in the number of platelets, hypoplasia, or absence of radial bone unilaterally or bilaterally. Hematologic, skeletal, cardiac (particularly tetralogy of Fallot and septal-atrial defects), and gastrointestinal anomalies are most commonly associated with TAR syndrome. Skeletal changes result in a higher risk of dental and craniofacial trauma in patients with the syndrome. Thus, it is important for the dentist to be aware of the characteristics of TAR syndrome and its clinical management for better care of these patients. The objective of this study is to describe a case report of a 26-year-old patient with TAR syndrome with a history of trauma and root fracture of tooth 11 and alveolar bone ridge. During anamnesis, root fractures requiring the extraction of the 11 tooth, alveolar bone ridge fracture in the adjacent region, and dental trauma were observed. A hematological evaluation and blood and radiological examinations were performed. Osseointegrated implant was performed using the guided surgery and flapless technique, as well as prosthetic rehabilitation in the affected region. This report discusses the importance of careful planning, such as the use of incisions and conservative surgery, techniques for alveolar ridge preservation, gingival manipulation, and prosthesis confection. The patient was attended by a hematologist throughout the treatment.

** Key words:**TAR syndrome, absent radii and thrombocytopenia, dental implants, oral surgery.

## Introduction

Thrombocytopenia-absent radius (TAR) is a rare congenital disease first described in 1929 and, subsequently, defined as a syndrome in 1969 (1,2). The frequency estimated was 0.42 cases per 100,000 live births (3). Clinically, the syndrome is characterized by thrombocytopenia associated with bilateral radial bone aplasia, and it may be associated with other diseases, such as hematological (platelet, white, and red blood cell), skeletal, urogenital, cardiac (particularly tetralogy of Fallot and septal atrial defects), and gastrointestinal disorders (2,3). The low rates of platelet counts are the most important hematological disorder related to TAR syndrome (2,3), and this fact may be a great concern for surgical treatments (4).

There are few studies in the literature that have related the clinical condition of patients diagnosed with TAR syndrome and surgical treatments involving bone rehabilitation for dental implants. Thus, the present study reports a clinical case of a patient diagnosed with TAR syndrome who underwent two surgical procedures following trauma caused by a car accident. The first surgery was a tooth extraction associated with bone graft for alveolar ridge preservation and buccal bone reconstitution; the second was a guided implantation surgery for dental rehabilitation.

## Case Report

The patient, a 26-year-old male non-smoker with a diagnosis of TAR syndrome, presented at the dental clinic for the treatment of dental fractures of teeth 21, 11, and 12 that resulted from trauma related to a car accident. Previous medical history was positive for TAR syndrome, with presence of thrombocytopenia at birth, phocomelia, and deformities of the upper and lower limbs. The patient reported recurrent episodes of thrombocytopenia and being in the care of a hematologist; he was also being treated for a fracture of the right femur as a result of the accident. No bleeding or blood transfusion had been reported. The patient did not report controlled drugs for continuous use, allergies, and adverse drug reactions. The evaluation by the medical clinic was carried out and the report did not show kidney dysfunction or other diseases that could prevent dental surgery. The patient agreed to sign an informed consent form, and the clinical case was conducted in a private practice according to the principles of the Declaration of Helsinki.

**Figure 1:**
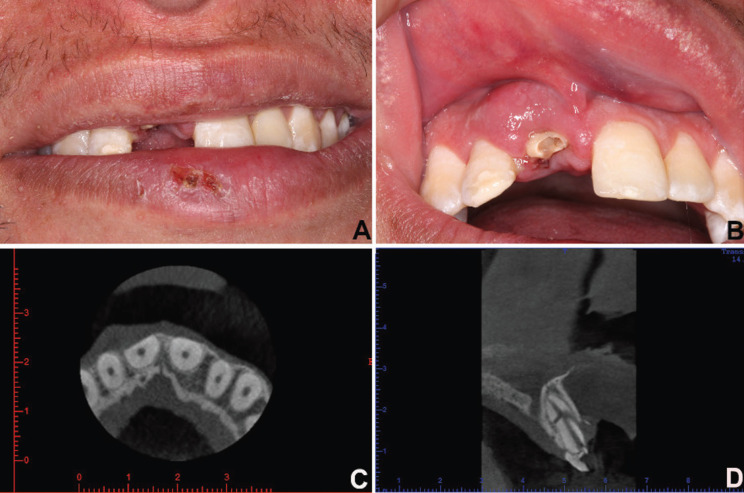
A) Abrasions and localized labial edema. B) Gingival edema and clinical crown fracture apparent without pulp involvement in teeth 11. clinical insertion. A cone beam computed tomography examination showed C) bone fractures and D) multiple root fractures.

**Figure 2:**
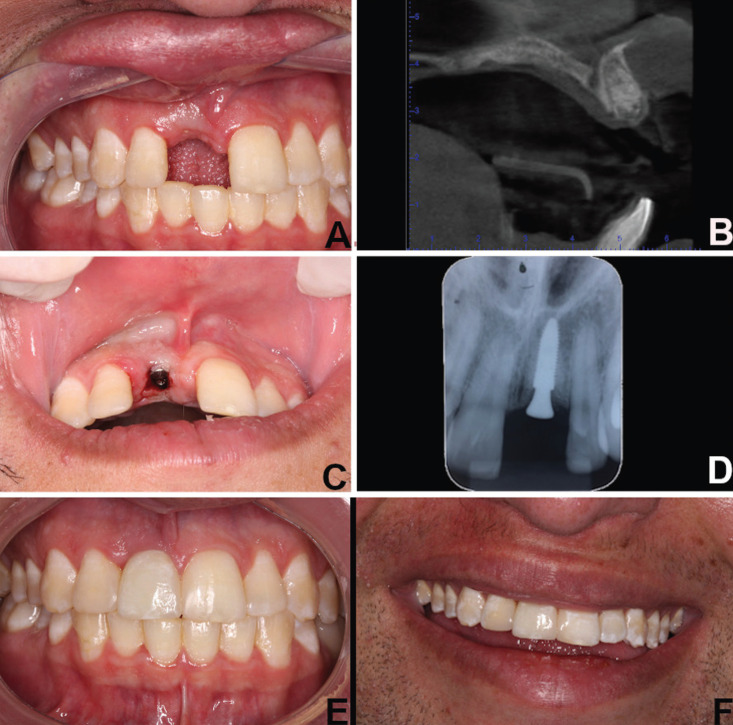
A) Clinical examination after suture removal. B) Axial section of cone beam computed tomography after the healing and complete osseointegration of the grafted biomaterial. C) Implant installation surgery and healing abutment placed simultaneously at the surgical procedure. D) Intraoral periapical radiographs showing implant osseointegrated. Intraoral E) and F) extraoral examination showing clinical appearance after permanent ceramic crown on tooth 11 and restoration on tooth 12 and 21.

Clinical examination revealed abrasions, localized labial and gingival edema, and clinical crown fracture apparent without pulp involvement in teeth 11 (Fig. 1A,B). Pulp vitality testing was positive for both teeth. We observed a low smile line (Fig. 1A) and thick gingival biotype, with no change in probing depth and no loss of clinical insertion. A cone beam computed tomography examination showed bone fractures observed in the axial sections (Fig. 1C) and multiple root fractures observed in the sagittal sections (Fig. 1D). Restorative treatment with composite resin for teeth 12 and 21 was proposed to the patient (Fig. 1B). Furthermore, the treatment plans included the extraction of tooth 11 with bone grafting for the alveolar ridge preservation and buccal bone reconstitution. Following this, an osseointegrated dental implant was planned for a second treatment. The graft technique selected was an insertion of lyophilized bovine bone associated at resorbable collagen membrane (4). During the surgical stages, a removable partial denture was used for provisional rehabilitation of tooth 11 and occlusal stabilization. For surgical planning, a complete blood count was performed; hematological exams showed leukopenia (global leukocytes 14,400/mm3) and an increased number of neutrophils (9,575/mm3) and lymphocytes (3,802/mm3). The platelet count was 96,000/mm3. The patient was evaluated by a hematologist who attended him in the dental surgical procedure. Preoperative medication consisted of dexamethasone 4.0 mg (one Tablet 12 hours before and one Tablet 1 hour before the procedure) and amoxicillin 500 mg (2 capsules, 1 hour before the procedure). Postoperatively, the patient was prescribed amoxicillin 500 mg, 1 capsule every 8 hours for 7 days, and sodium dipyrone 500 mg, one Tablet every 6 hours for pain. A conservative surgical technique was performed. For better visualization and flap coaptation, a smooth detachment was performed. Following the removal of the root fragments and fractured bone tissue, the bone defect was filled with 0.25–1mm-sized particles of xenogeneic graft (Bio-Oss®, Geistlich Pharma AG, Wolhusen, Switzerland) associated with bovine collagenous membrane (Lumina-Coat®, Criteria, São Paulo, Brazil). The surgical wound was sutured to prevent tension, and there was full coverage of the surgical area, protecting the graft and the membrane and promoting hemostasis. The sutures were removed after 15 days (Fig. 2A), and no adverse postoperative bleeding, pain, or edema events were observed. After six months, the healing and osseointegration of the grafted biomaterial were complete, and a new cone beam computed tomography was performed to plan the implant installation procedure (Fig. 2B). It was proposed to the patient that an osseointegrated implant be installed by guided surgery, without flap folding, through virtual planning. The patient was given new pre- and postoperative drug prescriptions and performed the same protocol as that for the first surgery. The surgery for the implant installation of 3.5 mm diameter and 11 mm length (Neodent/Straumann, Curitiba, Brazil) was performed with a flapless procedure, as planned, and the healing abutment was placed simultaneously at the surgical procedure (Fig. 2C). When four months had elapsed since the implant placement (Fig. 2D), the patient was subjected to gingival tissue manipulation with a temporary crown to improve aesthetics and to make the permanent ceramic crown (Fig. 2E,F).

The patient was monitored with periodic examinations. After the installation of the prosthesis, prophylactic procedures and clinical examination were performed every two months for the first six months and every four months after that period. The patient was instructed to use interdental cleaning devices (dental floss, interdental brushes, dental floss irrigator) and toothbrush. Patient remains under follow-up and is good health.

## Discussion

TAR syndrome is a rare and genetic disorder in most of cases autosomal recessive (1,2). Individuals affected suffer high rates of mortality in early life due to low platelet counts, which can result in severe bleeding (2,5); platelet production deficiency results from the failure of megakaryocytes to complete maturation in the bone marrow (5). Platelet counts gradually increase in childhood and tend to normalize in adulthood (2). However, they may vary throughout the individual’s life span and may change following infection, emotional stress, or some difficulty in the production and maturation of megakaryocytes (2). The study presents a patient diagnosed with TAR syndrome who submitted to oral rehabilitation with dental implants after suffering trauma in a car accident. In this case, changes in platelet count (96,000/mm3) were noted in a blood-count examination 30 days after the accident. The low levels of platelets can result in bleeding risks, which lead to the need for transfusion following surgical procedures (2). Therefore, it is necessary to perform a platelet-count examination prior to surgery, as well as to analyze clotting time using the international normalized ratio (INR) (2). It is important to emphasize the need for a complete assessment of the patient’s systemic status by a hematologist, which was demonstrated in the presented case.

Changes in white blood cell counts may also be noted following accidents. After the hematology tests, leukopenia was found to be present 30 days after the accident and was associated mainly with neutrophil levels. Similar to thrombocytopenia, the nature of leukopenia is transient and can be observed in patients suffering from this syndrome (2). The changes in neutrophil levels may have been related to systemic responses resulting from femoral trauma, abrasions, and fractures of dental roots’ suffered by the patient and, possibly, secondary infections at these sites.

A conservative extraction technique was performed in this case, with the removal of dental fragments and the cautious removal of tissue damaged by dentoalveolar trauma. The choice of the flapless technique in the reported case becomes interesting since the remaining structures were maintained without morbidity (6). Suturing was able to be performed without tensioning, and the coaptation of the edges allowed hemostasis and protection of the biomaterials so that they could perform their reparative function (7-9). The osseointegration period of the graft was respected (7,8). The choice of lyophilized bovine bone graft, using small particles, may be justified by its proven grafting efficacy for the purpose of alveolar preservation (7). The association with resorbable bovine collagen membrane is considered a safe graft technique for the purpose of alveolar preservation (7). These techniques are associated with the efficacy of biomaterials for the purpose of rehabilitation, reporting greater probabilities for the recovery of lost structures and a lower risk of additional surgeries needed for repair. The use of image-guided surgery to install the dental implant provided safety, and due to the possibility of surgery without the flap, there was a decrease in the risk of bleeding and invasion of important anatomical structures, such as the nasal cavity and incisor canal (6). The immediate installation of the healing abutment along with the implant enabled the exclusion of a second surgery to expose it after the osseointegration period, thereby reducing risks to the patient (10,11). The ceramic crown procedure was performed to ensure adequate interproximal contact and a well-defined emergence profile in order to obtain not only aesthetics but also gingival stability.

TAR syndrome and other hematological disorders of patients, such as hemophilia and the continuous use of anticoagulants, require that any dental intervention include preventive measures and meticulous planning regarding the procedures to be performed. The accurate interpretation of hematology exams, as well as the use of less-invasive procedures and a multidisciplinary approach should be used routinely for these patients, thereby promoting clinical safety related to dental care.
